# An Extremely Preterm Infant Born at 23 Weeks' Gestation With an Interrupted Aortic Arch Complex: A Case Report

**DOI:** 10.7759/cureus.41389

**Published:** 2023-07-05

**Authors:** Mitsuhiro Haga, Kanako Itoh, Akio Ishiguro, Yoichi Iwamoto, Takuro Kojima, Satoshi Masutani

**Affiliations:** 1 Department of Pediatrics, Saitama Medical Center, Saitama Medical University, Kawagoe, JPN; 2 Department of Pediatric Cardiology, Saitama Medical University International Medical Center, Kawagoe, JPN

**Keywords:** hypoxic pulmonary vascoconstriction, 22q11.2 deletion, congenital aortic arch anomaly, extremely low birth weight infant, complex congenital heart disease

## Abstract

We present a case of an infant male born at 23 weeks' gestation with an interrupted aortic arch (IAA) complex. We treated the patient with hypoxic gas ventilation to address developing systemic undercirculation in the acute postnatal phase. As the symptoms of bronchopulmonary dysplasia evolved, hypoxic gas ventilation was no longer required to stabilize the hemodynamics. The patient was discharged home after undergoing the palliative surgical procedure of bilateral pulmonary artery banding and ductus arteriosus stent implantation. Although he suffered from pulmonary hypertension, it was controllable with oxygen supplementation and pulmonary vasodilators. There are limited therapeutic options available for extremely preterm infants with critical congenital heart defects (CHDs). Hypoxic gas ventilation might be considered as one of the options, with its risks taken into account, to manage extremely preterm infants with CHDs with pulmonary overcirculation before performing surgical interventions.

## Introduction

Managing preterm infants with critical congenital heart defects (CHDs) is a major challenge for healthcare providers [1,2]. Available therapeutic options are limited due to technical difficulties in surgical interventions and complications associated with premature birth [1,2]. Clinical reports of extremely preterm infants [<28 weeks of gestational age (GA)] with critical CHDs are scarce, and there is no consensus on the optimal therapeutic strategy. We discuss a case of an extremely preterm infant born at 23 weeks of GA with an interrupted aortic arch (IAA) complex. We treated him with hypoxic gas ventilation to stabilize the hemodynamics in the acute postnatal phase. The patient underwent palliative surgery at 147 days of life and survived and was discharged home.

## Case presentation

**Figure 1:**
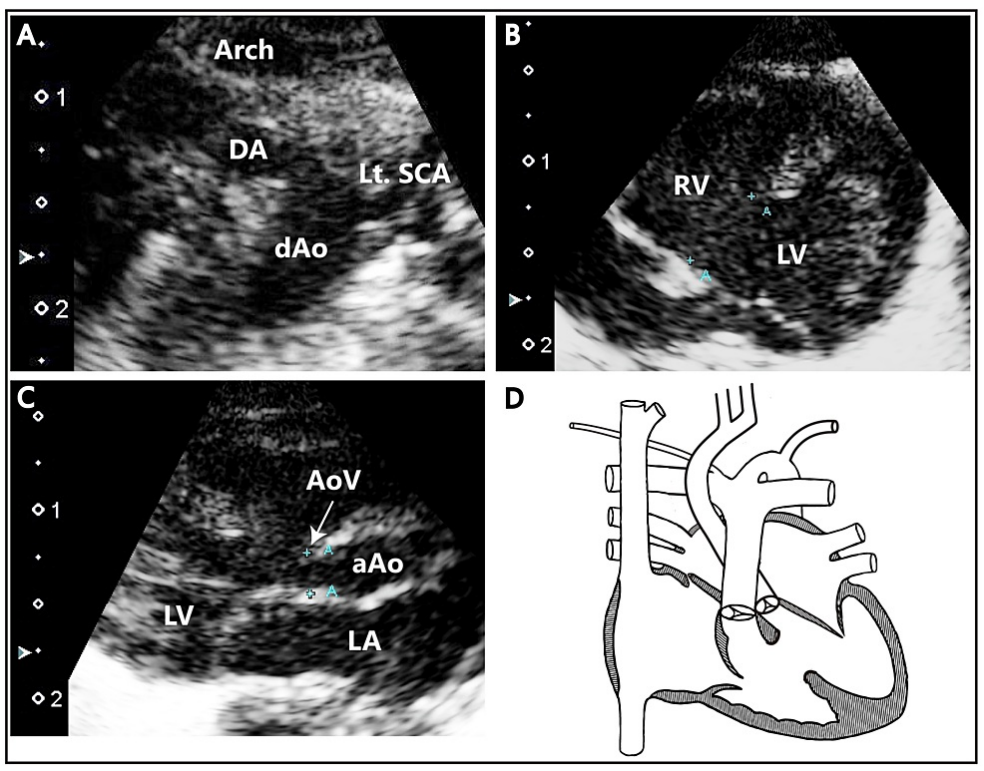
Transthoracic echocardiography images and a schematic diagram of the patient’s heart (A) Suprasternal long-axis view. The aortic arch is interrupted proximally to the left subclavian artery. The ductus arteriosus provides blood flow to the descending aorta. (B) Apical four-chamber view. A perimembranous ventricular septal defect with a maximum diameter of 4.8 mm can be observed. (C) Left parasternal long-axis view. A narrowed aortic valve can be seen. The maximum diameter of the aortic valve is 2.2 mm. (D) Illustration of the structure of the heart

Our patient's mother was a 19-year-old primigravida Asian woman. No abnormality had been found in fetal ultrasonography screening. At 23 weeks and three days of gestation, the mother had been emergently transferred to our hospital due to threatened preterm labor. She had been diagnosed with placental abruption and sent for an emergency caesarian section. She had subsequently delivered a male neonate with a birth body weight of 545 g. After admission, the infant was diagnosed with IAA type B (Celoria-Patton classification), perimembranous ventricular septal defect, and aortic valve stenosis on echocardiography (Figure 1).

The percutaneous oxygen saturation (SpO_2_) levels were not different between the right upper arm and other extremities, suggesting that the patient had an aberrant right subclavian artery. Contrast-enhanced CT performed at term-equivalent age confirmed the aberrant right subclavian artery originating from the right pulmonary artery. Genetic testing led to a diagnosis of 22q11.2 deletion syndrome.

**Figure 2:**
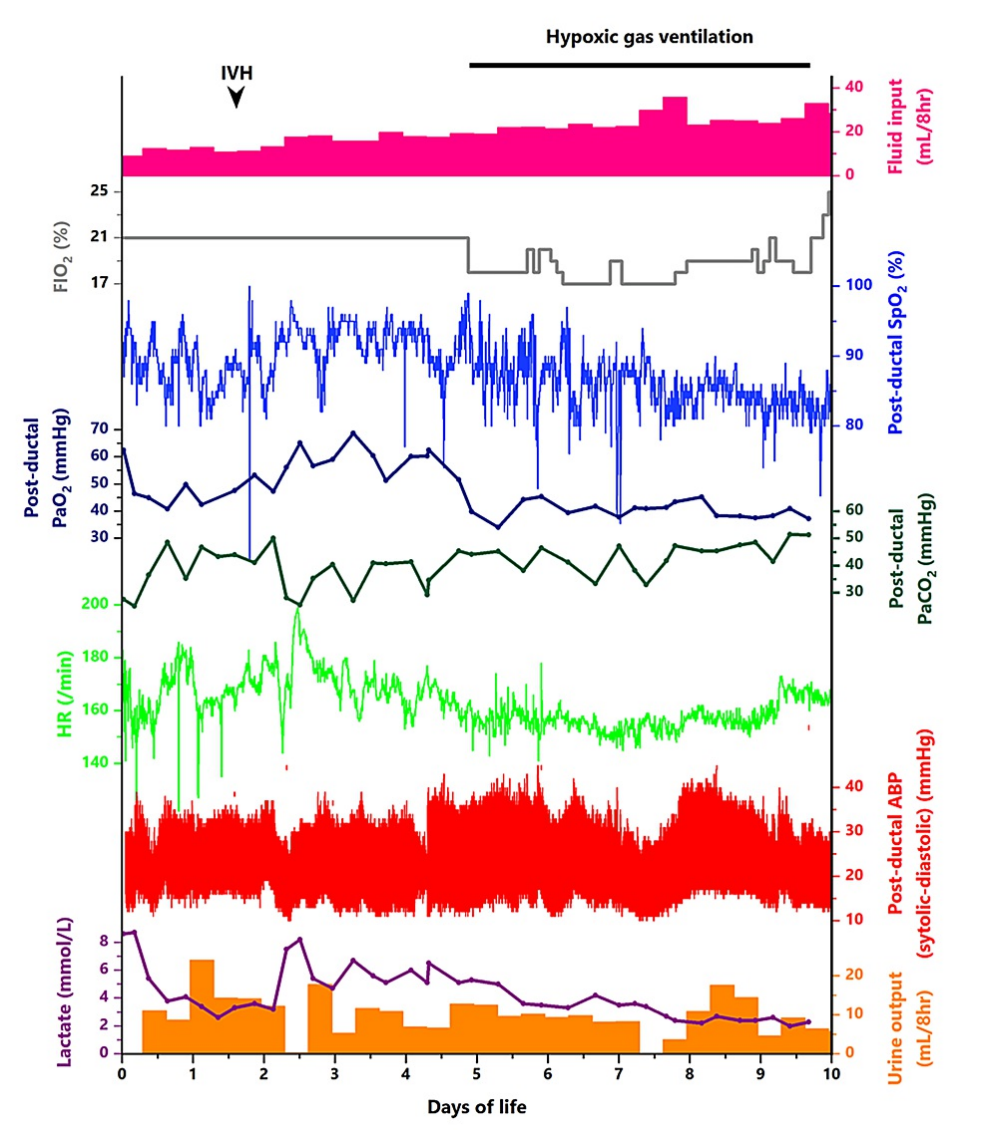
The patient’s clinical course from admission to 10 days of life The patient developed intraventricular hemorrhage (IVH) at two days of life (arrowhead). His heart rate (green line graph) and lactate level (purple line graph) increased after the development of IVH. Transfusions of red blood cells and fresh frozen plasma restored the heart rate to the normal range, but the lactic acidosis was unresolved. The post-ductal percutaneous oxygen saturation (SpO_2_) level (blue line graph) increased to 95% with a fraction of inspiratory oxygen (FiO_2_) of 21% at five days of life, suggesting the development of pulmonary overcirculation and systemic undercirculation. Hypoxic gas ventilation with nitrogen gas supplementation was started on the same day (gray line graph). The post-ductal partial arterial pressure of oxygen (PaO_2_) decreased from around 60 mmHg to around 40 mmHg (dark blue line graph). We controlled the post-ductal partial arterial pressure of carbon dioxide (PaCO_2_) to around 40 mmHg (dark green line graph). Although the post-ductal arterial blood pressure (red floating bar graph) and urine output (orange bar graph) did not change, the lactate levels (purple line graph) decreased as the SpO_2_ levels gradually decreased. At nine days of life, the required FIO_2_ increased, and the continuous inspiration of hypoxic gas was discontinued. Note that whole blood samples from the post-ductal arterial line were analyzed for PaO_2_, PaCO_2_, and lactate levels using an automated blood gas analyzer (ABL90 FLEX PLUS, Radiometer, Copenhagen, Denmark) ABP: arterial blood pressure; FiO_2_: a fraction of inspiratory oxygen; HR: heart rate; IVH: intraventricular hemorrhage; PaO_2_: partial arterial pressure of oxygen; PaCO_2_: partial arterial pressure of carbon dioxide; SpO_2_: percutaneous oxygen saturation

After the diagnosis of the IAA complex, continuous infusion of lipo-prostaglandin E1 was administered. The ductus arteriosus (DA) remained patent with a 2.0-2.5 mm diameter with a low-dose infusion of lipo-prostaglandin E1 (0.5 ng/kg/min) until the palliative operation. The neonate developed an intraventricular hemorrhage (IVH) at two days of life. The bleeding was observed in both lateral ventricular spaces without causing distention (Papile grade of severity of IVH was II out of IV) [3]. As a result of IVH-related blood loss, the infant experienced circulatory collapse with tachycardia and had elevated lactate levels (Figure 2).

We resuscitated the patient with the transfusion of blood products, but the arterial lactate levels remained elevated at 4-7 mmol/L. At five days of life, the post-ductal SpO_2_ level increased to 95% under a fraction of inspiratory oxygen (FiO_2_) of 21%. Pulmonary venous congestion became evident on chest radiography. Echocardiography showed an increased left-to-right shunt blood flow through the DA compared to the findings on admission. We evaluated that he had developed pulmonary overcirculation and systemic undercirculation due to the postnatal decrease in pulmonary vascular resistance. We set the target post-ductal arterial partial pressure of carbon dioxide (PaCO_2_) level at 40-50 mmHg. However, given the patient’s metabolic acidosis, we were cautious about inducing further hypercapnia, which could lead to severe acidemia. To prevent the deterioration of hemodynamics, we decided to initiate hypoxic gas ventilation therapy.

**Figure 3:**
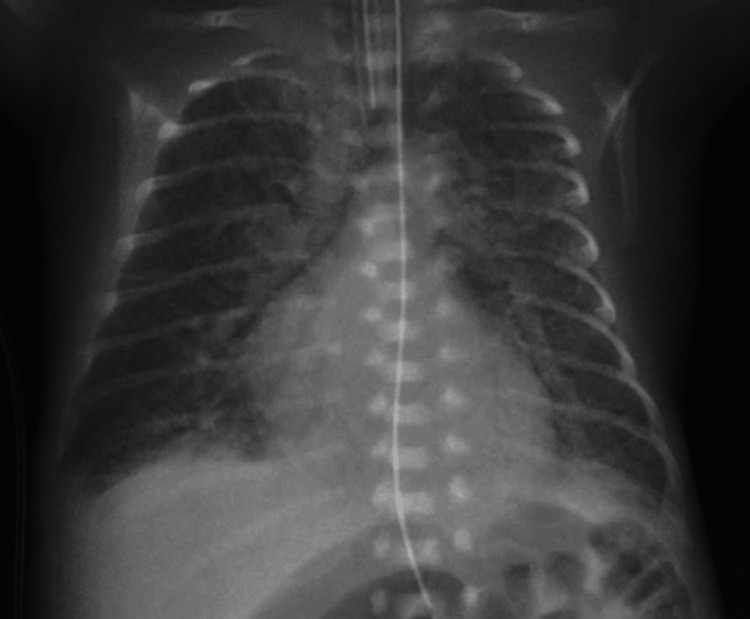
Chest X-ray at 10 days of life The chest X-ray shows irregular opacities in both lung fields, and the borders of the heart and the diaphragm are obscured

In Japan, hypoxic gas ventilation therapy for infants with CHDs with increased pulmonary blood flow is an authorized treatment regulated by the Japanese government, and our institution did not require the ethical committee’s approval to administer this therapy to the patient. After obtaining written informed consent from the parents, we initiated hypoxic gas ventilation with nitrogen gas supplementation. Because we could not monitor the pre-ductal SpO_2_ levels due to the aberrant right subclavian artery, and because we took into serious consideration the fact that the patient was extremely preterm, we tentatively set the target range of post-ductal SpO_2_ at 80%-90%, which is a much milder target than generally used [4], to avoid excessive hypoxemia. We diligently monitored various parameters during hypoxic gas ventilation therapy, including vital signs, urine output, post-ductal arterial partial pressure of oxygen (PaO_2_), PaCO_2_, and ultrasonography findings. Following the initiation of hypoxic gas ventilation, there was a decrease in post-ductal PaO_2_ levels from approximately 60 to around 40 mmHg. Ultrasonography of DA blood flow showed that the velocity-time integral ratio of right-to-left and left-to-right blood flow was restored after hypoxic gas ventilation. Although the post-ductal blood pressure and urine output did not change, the lactate levels decreased as the post-ductal SpO_2_ levels decreased toward the target range (Figure 2). At nine days of life, the continuous inspiration of the subatmospheric oxygen level was no longer required to maintain the target SpO_2_ range. We assessed that the evolving bronchopulmonary dysplasia (BPD) accounted for the worsening oxygenation with chest radiography findings of irregular opacities in the lung field (Figure 3).

At 10 days of age, the patient developed refractory hypotension that did not respond to catecholamines and volume expanders. Administration of hydrocortisone effectively restored blood pressure and urine output, leading us to suspect adrenal insufficiency of prematurity as the underlying cause of the hypotension. At 13 days of life, we identified a hematoma in the right parietal region with cranial ultrasonography. The hematoma measured 7.4 mm in maximum width and exerted pressure on the right parietal lobe from outside the brain. The most probable diagnosis was an acute subdural hematoma. A fresh frozen plasma transfusion was administered to restore normal coagulation status. A craniotomy was deemed unnecessary as there were no signs of brain herniation. The exact cause of the hemorrhage remained unknown as the patient had no history of traumatic head injury. The bleeding tendency due to prematurity and 22q11.2 deletion syndrome may have contributed to the development of the hematoma. MRI at term-equivalent age showed porencephaly in the right parietal lobe. The enteral feeding volume was cautiously increased due to signs of paralytic ileus on abdominal X-rays. It took 52 days of life to achieve an enteral feeding volume of 100 mL/kg/day. The patient did not exhibit any signs of definite necrotizing enterocolitis, such as bloody stools or pneumatosis intestinalis. We weaned him off mechanical ventilation at 71 days of life. He underwent palliative surgery of bilateral pulmonary artery banding with 2.5 mm width of polytetrafluoroethylene strips and DA stent implantation at 147 days of life when his body weight was 2.3 kg. He was discharged home after the surgery.

Cardiac catheterization at seven months of life revealed pulmonary hypertension (PH) with mean pressures of 21 and 27 mmHg in the right and left pulmonary arteries, respectively. Home oxygen supplementation therapy and pulmonary vasodilators were started. Follow-up catheterization at 26 months of age showed a significant improvement in PH; the mean pressures of the right and left pulmonary arteries were 14 and 18 mmHg, respectively.

The patient is 32 months old at the time of writing this report and is currently on home oxygen therapy of 1.0 L/min of flow from the nasal cannula and pulmonary vasodilators tadalafil and macitentan. He suffers from severe developmental delay; he is able to sit without support but cannot walk independently. Total correction surgery was declined by the parent due to the risks of the procedure.

## Discussion

To the best of our knowledge, our patient is the most premature infant with an IAA complex who survived to be discharged home. Hypoxic gas ventilation stabilized the hemodynamics of this patient in the acute postnatal phase. Evolving bronchopulmonary dysplasia appeared to have contributed to maintaining the balance of systemic and pulmonary blood flow in the subacute to chronic phases, and we were able to perform palliative surgery after sufficient body weight was gained.

To review the previous case reports of critical CHDs in infants born extremely preterm, we searched the databases PubMed® and EMBASE®. We found 14 cases of extremely preterm infants with critical CHDs other than patent DA from 2008 to 2022 (Table 1) [5-16]. This literature survey showed an apparent gap in postnatal days for the first interventions depending on infants’ GA. The median days of life at the first surgical intervention was 36 in four cases of infants born at 23-25 weeks of GA, whereas it was 11 in 10 cases of those born at 26-27 weeks. Although this difference could not be evaluated with any statistical test due to the small sample size and heterogeneity in groups, it would be conceivable that the prematurity of infants born before 26 weeks of GA led to delaying the surgical interventions before the infants’ condition stabilized.

**Table 1:** Case reports of critical congenital heart defects in extremely preterm infants AP window: aortopulmonary window; BPD: bronchopulmonary dysplasia; BW: birth body weight; CoA: coarctation of the aorta; DA: ductus arteriosus; dTGA/IVS: dextro-transposition of the great arteries with an intact ventricular septum; dTGA/VSD: dextro-transposition of the great arteries with ventricular septal defect; FIP: focal intestinal perforation; GA: gestational age; MAPCA: major aortopulmonary collateral arteries; NA: not available; PA: pulmonary artery; PA/VSD: pulmonary atresia with ventricular septal defect; PVS: pulmonary valve stenosis; TTTS: twin-to-twin transfusion syndrome; ROP: retinopathy of prematurity; VSD: ventricular septal defect

Authors	GA (weeks)	BW (g)	Diagnosis	First interventions		Reported underlying conditions	Reported complications
				Day of life	Procedures		
Holzer et al. [5]	24	680	PVS	25	Transventricular balloon pulmonary valvuloplasty	None	None
Wirbelauer et al. [6]	26	545	CoA	9	End-to-end anastomosis surgery	Discordant twin	None
Bialkowski et al. [7]	25	740	CoA	26	DA clipping	TTTS	ROP, BPD, phrenic nerve palsy
Godown et al. [8]	25	520	Hemitruncus	45	DA clipping	None	Pulmonary hypertension
Rios et al. [9]	27	1,010	dTGA/IVS	0	Balloon atrial septostomy	None	None
Kido et al. [10]	27	912	VSD	9	PA banding + DA ligation surgery	None	None
	26	904	dTGA/VSD	13	PA banding + DA ligation surgery	None	West syndrome
	23	626	AP window	64	Bilateral PA banding surgery	None	Hydrocephalus
Stegeman et al. [11]	27	NA	CoA	12	Stent implantation	None	Aortic aneurysm
Ide et al. [12]	27	810	PA/VSD + MAPCA	2	DA banding surgery	None	FIP
Shetty Shantharam et al. [13]	27	506	CoA	21	End-to-end anastomosis + DA clipping surgery	Discordant twin	BPD
Takeoka et al. [14]	27	556	CoA	57	End-to-end anastomosis surgery	TTTS donor	None
Tomotaki et al. [15]	27	700	AP window	7	AP window ligation + DA ligation	None	None
Al-Ammouri et al. [16]	26	590	CoA	14	Balloon angioplasty	TTTS donor	None
Present case	23	545	IAA complex	147	PA banding + DA stent implantation	22q11.2 deletion syndrome	Intracranial hemorrhage, porencephaly, BPD

The most critical problem in infants with CHDs in the acute postnatal phase is the dynamic change in the pulmonary and systemic blood flow balance. Pulmonary vascular resistance rapidly decreases after birth, which can deteriorate the hemodynamics in patients with CHDs with pulmonary overcirculation. The primary objective of hypoxic gas ventilation is to maintain a high pulmonary vascular resistance, countering the physiological decrease that occurs in the postnatal period. This therapy offers two potential benefits, as supported by theoretical considerations. Firstly, it aims to ensure sufficient blood flow to vital organs, preventing compromised oxygen delivery, even with a modest reduction in peripheral oxygen saturation. Secondly, it aims to reduce the workload on the heart by decreasing the pulmonary-to-systemic blood flow ratio.

Several clinical studies have examined the effectiveness of hypoxic gas ventilation in neonates with hypoplastic left heart syndrome, IAA, and coarctation of the aorta [17-19]. These studies have shown promising results, indicating that hypoxic gas ventilation can improve oxygen tissue supply by maintaining a high pulmonary vascular resistance in these patient populations. While no large-scale randomized controlled trials have been conducted to evaluate the treatment, a prospective multicenter observational study in Japan has provided valuable insights. This study demonstrated an increase in urine output following the initiation of hypoxic gas ventilation without any adverse events, such as severe brain damage and PH, at one year of age [20]. Although further research is needed to establish the efficacy and long-term outcomes of hypoxic gas ventilation, these initial findings are encouraging and suggest the potential benefits of this treatment approach. Hypoxic gas ventilation was authorized for treating infants with CHDs with increased pulmonary blood flow by the government in Japan in 2018. Consequently, it has become widely available at Japanese institutions. We chose to employ hypoxic gas ventilation other than hypercapnic therapy in our patient for two reasons. Firstly, we anticipated that intensified hypercapnic therapy could lead to severe acidemia due to mixed acidosis in this patient. Given the immaturity of renal tubular function in neonates born before 24 weeks of gestation, metabolic acidosis was inevitable [21]. Secondly, sedative drugs and muscular relaxants, which are often required to suppress spontaneous breathing during hypercapnic therapy, can cause feeding intolerance.

There is no report of hypoxic gas ventilation for extremely preterm infants with complex CHDs in the literature. We applied this therapy to the present case with the utmost caution when the patient showed signs of pulmonary overcirculation. To avoid acute exacerbation of PH, we set the target range of post-ductal SpO_2_ at 80%-90%, which was milder than the recommended range of pre-ductal SpO_2_ of 75%-80% during hypoxic gas ventilation for single ventricle defects [4]. Lactate levels decreased as the post-ductal SpO_2_ decreased toward the target range, suggesting tissue oxygen delivery improvement. We assessed that the increase in systemic blood flow by restoring pulmonary vascular resistance surpassed the decrease in arterial blood oxygen content. Our patient developed severe intracranial hemorrhage three days after hypoxic gas ventilation was terminated. The primary cause of the intracranial hemorrhage was likely the prematurity of the patient.

Contrary to our expectations, the patient required hypoxic gas ventilation to stabilize the hemodynamics only for five days. High pulmonary vascular resistance associated with evolving BPD must have contributed to maintaining a balance in pulmonary and systemic blood flow from the subacute to the chronic phase in this patient. The lung in infants born before 24 weeks of GA is still in between the canalicular and early saccular phase with a smaller pulmonary vascular bed than that of term neonates [22]. Various insults after birth, such as mechanical ventilation, oxygen exposure, and pulmonary edema, result in arrested lung development and dysregulated microvascular growth [23]. The more premature the infants are at birth, the higher the risk of developing PH from the early stage of life [24]. Fortunately, early PH from evolving BPD appeared to have worked optimally in this patient. Given the risk of developing an irreversible pulmonary vascular occlusive disease, we set the target range of post-ductal SpO_2_ at 80%-90% to avoid severe hypoxemia. Although almost five months had elapsed before the patient underwent the first palliative surgery, the pulmonary vascular disease was not severe and was controllable with oxygen supplementation and pharmaceutical treatment.

## Conclusions

Surgical interventions will have to be delayed in infants with critical CHDs born before 26 weeks of GA due to their extreme prematurity. Hypoxic gas ventilation might be considered as an option, with its risks taken into consideration, for stabilizing hemodynamics before surgical interventions in extremely preterm infants with CHDs with pulmonary overcirculation.
